# Supplementary material for: Skeletal anatomy of the early Permian parareptile Delorhynchus with new information provided by neutron tomography
Source: PeerJ. 2023 Aug 22;11:e15935. doi: 10.7717/peerj.15935 (PMC10452630; doi:10.7717/peerj.15935)
Supplement: Supplemental Information 2 — Each character for the phylogeny in this study, and the indication of changes where necessary as well as the source of each character where necessary. [file peerj-11-15935-s002.docx]

APPENDIX 1

REFERENCES

CIS refers to: Cisneros JC, Kammerer CF, Angielczyk KD, Frobisch J, Marsicano C, Smith RMH, Richter M. 2020. A new reptile from the lower Permian of Brazil (*Karutia fortunata* gen. et sp. nov.) and the interrelationships of Parareptilia. *Journal of Systematic Paleontology*, 18:1939-1959, DOI: 10.1080/14772019.2020.1863487

DEB96 refers to: deBraga M. & Reisz RR. 1996. The Early Permian reptile *Acleistorhinus pteroticus* and its phylogenetic position. *Journal of Vertebrate Paleontology*, 16:384-395. DOI: 10.1080/02724634.1996.10011328

DEB97 refers to: deBraga M, Rieppel O. 1997. Reptile phylogeny and the interrelationships of turtles. *Zoological Journal of the Linnean Society*, 120:281-354

LAU refers to: Laurin M, Reisz RR. 1995. A reevaluation of early amniote phylogeny. *Zoological Journal of the Linnean Society*, 113:165-223

LEE95 refers to: Lee MSY. 1995. Historical burden in systematics and the interrelationships of ‘parareptiles’. *Bioogical Reviews* 70: 459–547.

LEE97 refers to: Lee MSY. 1997. Pareiasaur phylogeny and the origin of turtles. Zoological Journal of the Linnean Society 120: 197¬–280.

MAC refers to: MacDougall MJ, Scott DS, Modesto SP, & Reisz RR. (2017) New material of the reptile *Colobomycter pholeter* (Parareptilia: Lanthanosuchoidea) and the diversity of reptiles during the Early Permian (Cisuralian). *Zoological Journal of the Linnean Society*, 180:661-771.

MOD99 refers to: Modesto SP. 1999. Observations on the structure of the Early Permian reptile *Stereosternum tumidum* Cope. *Palaeontologica Africana* 35:7–19.

MOD15 refers to: Modesto SP, Scott DM, MacDougall MJ, Sues H-D, Evans DC, Reisz RR. (2015) The oldest known parareptile and the early diversification of reptiles. *Proc. R. Soc. B* 282:20141912

MUL refers to: Muller J, Tsuji LA. (2007) Impedance-matching hearing in Paleozoic reptiles: evidence of advanced sensory perception at an early stage of amniote evolution. *PloS ONE* 2:e889 https://doi.org/10.1371/journal.pone.0000889

REI07 refers to: Reisz RR, Müller J, Tsuji L, Scott D. 2007. The cranial osteology of *Belebey vegrandis* (Parareptilia: Bolosauridae), from the Middle Permian of Russia, and its bearing on reptilian evolution. *Zoological Journal of the Linnean Society* 151: 191–214.

REI09 refers to: Reisz RR, Godfrey SJ, Scott DM. (2009). *Eothyris* & *Oedalops*: do these early Permian synapsids from Texas and New Mexico form a clade? *Journal of Vertebrate Paleontology*, 29:39-47 DOI:10.1671/039.029.0112

List of characters used in the phylogenetic analysis:

(1) Number of premaxillary tooth positions: 0-3 (0); 4-6 (1); more than 6 (2). (CIS #166, MAC #168, MOD15 #168)

(2) Premaxillary contribution to lower margin of external naris in lateral view: nearly half of the margin or more (0); minimal or absent (1). (CIS #177)

(3) Greatly enlarged tooth on premaxilla: absent (0); present (1). (CIS #178)

(4) Snout shape: wider than tall (0); taller than wide (1). (CIS #173)

(5) Snout length: long (0); medium (1); short (2); very short (3). (CIS #180)

(6) Septomaxilla: curled in external naris (0); forming a sagittal wall that contacts the nasal (1); forming a sagittal wall, no nasal contact (2); splint-like, confluent with skull roof, no sagittal wall (3); absent (4). (CIS #179, modified from REI09 #10)

(7) Number of maxillary tooth positions: 0-15 (0); 16-24 (1); 25-29 (2); 30 or more (3). (Modified from CIS #165, MAC #167, MOD15 #167)

(8) Anterodorsal process of the maxilla: absent (0); present (1). (CIS #29, MAC #29, LAU #19)

(9) Anterolateral shelf of the maxilla: absent or minimal (0); massive (1). This character refers to an anterior extension of the anterodorsal maxillary process, which contributes to the posterodorsal border of the external nares. [New character]

(10) Anterior lateral maxillary foramen: equal in size to other maxillary foramina (0); much larger than other foramina (1). (CIS #30, MAC #30, LAU #20)

(11) Presence of size related heterodonty on the anterior 2/3 of the maxilla: present (0); absent (1). (CIS #36, MAC #35, LAU #24) Refers to teeth on the anterior 2/3 of the maxilla with a base width at least 50% larger than other teeth, as stated in MacDougall et al. (2017).

(12) If size related heterodonty is present on the maxilla, it is represented by: one tooth (0); two or more teeth (1). (CIS #37, MAC #37, LAU #25)

(13) Fluting on upper marginal teeth: absent (0); present (1). (CIS #181)

(14) Maxilla overhangs tooth row: absent (0); present (1). (CIS #174)

(15) Exposition of the narial opening: lateral (0); anterior (1). (CIS #28, MAC #29, LAU #19)

(16) Contribution of maxilla to external naris: maxilla is either excluded from naris or forms only its ventral/posterior edge (0); maxilla extends also to the posterodorsal margin of naris (1). (CIS #34, MAC #34, MUL #137)

(17) Narial shelf: absent (0); present (1). (CIS #1, MAC #1, modified from LAU #1)

(18) If narial shelf is present, it is: found on the nasal (0); found on the nasal and maxilla (1). (CIS #2, MAC #2, modified from LAU #1)

(19) Lateral exposure of lacrimal: single lateral exposure (0); double lateral exposure (1). [New character]

(20) Lacrimal contact with external naris: present (0); absent (1). (Modified from CIS #15, MAC #15, LAU #9)

(21) Foramen orbitonasale: absent (0); present (1). (CIS #16, MAC #16, modified from LAU #10)

(22) If foramen orbitonasale is present, it is: represented by a medial indentation on the lacrimal and a dorsal indentation on the palatine (0); enclosed between prefrontal, lacrimal and palatine (1). (CIS #17, MAC #17, modified from LAU #17)

(23) Dermal sculpturing: absent (0); present (1). (CIS #53, MAC #54, modified from LAU #38)

(24) Sculpturing involving circumorbital bumps: no distinctive ornamentation (0); circumorbital tubercles (1). (CIS #54, MAC #56, modified from LAU #38)

(25) Pits on skull roof: absent (0); present (1). (CIS #182)

(26) Ridges on cranial roof sculpturing: anastomosed (including “honey-comb pattern”) (0); weak or absent (1). (CIS #183)

(27) Contact between maxilla and prefrontal: absent (0); present (1). (CIS #33, MAC #33, MUL #136)

(28) Prefrontal-palatal contact: absent (0); present (1). (CIS #11, MAC #11, modified from LAU #6)

(29) If prefrontal-palatal contact is present, it is: narrow and acuminate (0); strong, with sutural base (1). (CIS #12, MAC #12, modified from LAU #6)

(30) Prefrontal medial flange: narrow (0); wide (1). (CIS #13, MAC #13, modified from LAU #7) A narrow prefrontal medial flange is at most 1/5^th^ of the orbital height, as in MacDougall et al. (2017).

(31) Prefrontal orbital spur: absent (0); present (1). (CIS #14, MAC #14, LAU #8)

(32) Prefrontal-postfrontal contact in dorsal view: present (0); absent (1). (CIS #3, MAC #3, modified from LAU #2).

(33) Postfrontal posterior border: does not reach the pineal foramen (0); contacts or falls within level of pineal foramen (1) surpasses posterior border of pineal foramen (2). (CIS #187)

(34) If prefrontal-postfrontal contact is absent, the frontal contribution to the orbital margin in dorsal view is: narrow (0); broad (1). (CIS #4, MAC #4, modified from LAU #2) The frontal occupies less than 1/3^rd^ of the orbital margin when the contribution is narrow, as mentioned in MacDougall et al. (2017)

(35) Frontal anterior process length: short (0); long (1); very long (2). (CIS #175)

(36) Frontal lateral lappet: absent (0); present (1). (CIS #5, MAC #5, modified from DEB96 #7)

(37) Postfrontal morphology in dorsal view: pseudo-rectangular (0); triradiate (1); thin and elongated, with a main oblique axis (2); strongly recurved (3). (CIS #188)

(38) Pineal foramen position: in the middle of the body of the parietal (0); displaced posteriorly (1); displaced anteriorly (2). (CIS #6, MAC #6, modified from DEB97 #49)

(39) Postparietal: present (0); absent (1). (CIS #7, MAC #7, modified from LAU #4)

(40) If postparietal is present, it is: paired (0); median (1). (CIS #8, MAC #8, modified from LAU #4)

(41) If postparietal is present, its size is: large (0); small (1). (CIS #9, MAC #9, modified from LAU #4) As in MacDougall et al. (2017), the postparietal is large when its width is equal to one parietal.

(42) If postparietal is present, it is positioned such that it is: dorsally exposed, integrated into skull table (0); occipital (1). (CIS #10, MAC #10, LAU #5)

(43) Suture between jugal and maxilla: straight, jugal thins out smoothly towards anterior direction (0); ‘stepped’, anterior most tip of jugal very narrow but expands broadly posteriorly along with a dramatic thinning of the posterior process of the maxilla (1). (CIS #32, MAC #32, MUL #133)

(44) Jugal anterior process: does not extend to anterior orbital rim (0); extends at least to level of anterior orbital rim (1). (CIS #18, MAC #18, LAU #11)

(45) Suborbital ramus of jugal: dorsoventrally broad (0); slender or reduced with no dorsoventral extension (1). (CIS #19, MAC #19) A slender suborbital ramus of the jugal has a dorsoventral height less than the suborbital portion of the maxilla, as per MacDougall et al. (2017).

(46) Posterior extension of jugal that contributes to temporal region of skull roof: present (0); absent (1). (CIS #20, MAC #20, MUL #131)

(47) Posterior edge of jugal: convex, rugose (0); straight or concave, rugose edge (1); concave, smooth edges (2); smooth, free edge. [New character]

(48) Postorbital morphology: small, occupies approximately one-third of dorsal orbital rim, not transversely broad and has approximately flat or convex dorsolateral surface (0); dorsolateral surface concave (recessed between orbit and temporal fenestra) (1); long and broad forming prominent supraorbital shelf (2); strongly recessed posterolateral surface forming anterior part of fossa around temporal fenestra (3). (CIS #170)

(49) Postorbital bar thickness: broad (0); narrow (1). (CIS #171)

(50) Postorbital posterior process: present (0); absent (1). (CIS #21, MAC #21, modified from DEB96 #14)

(51) Postorbital extension of orbit: absent (0); present (1). (CIS #52, modified from MAC #53, LAU #37)

(52) Postorbital-supratemporal contact: present (0); absent (1). (CIS #22, MAC #22, modified from LAU #12)

(53) Posterolateral corner of skull roof; formed by tabular (0); formed mostly by supratemporal (1); formed by parietal and small supratemporal or parietal alone (2). (CIS #23, MAC #23, LAU #15)

(54) Tabular: present (0); absent (1). (CIS #24, MAC #24, modified from LAU #17)

(55) If tabular is present, it is: part of skull table (0); largely occipital (1). (CIS #25, MAC #25, modified from LAU #17)

(56) Supratemporal: present (0); absent (1). (CIS #26, MAC #26, modified from LAU #18)

(57) If supratemporal is present, it is: large (0); small (1). (CIS #27, MAC #27, modified from LAU #18) The mediolateral width of this element, when small, is at most 1/10^th^ of the midline of the skull length as in MacDougall et al. (2017)

(58) Squamosal and post-temporal fenestra: separated (0); in contact (1). (CIS #38, MAC #38, LAU #26)

(59) Quadratojugal shape: does not reach beyond the level of the ventral orbital margin (0); extends dorsally beyond the level of ventral orbital margin (1). (CIS #39, MAC #39, modified from LAU #28)

(60) Maxilla and quadratojugal: in contact (0); separated (1). (CIS #31, MAC #31, LAU #22)

(61) Quadratojugal anterior extent: reaches posterior border of orbit (0); does not reach level of posterior border of orbit (1). (CIS #40, MAC #40, modified from LAU #23)

(62) Quadratojugal dorsal process under skull roof: absent (0); present, extending high up under adductor chamber (1). [New character]

(63) Quadratojugal ornamentation: confluent with the cheek and not ornate in any manner (0); ornamented, dermal protuberances project from its surface (1). (CIS #41, MAC #41, DEB97 #43)

(64) Upper temporal fenestra: absent (0); present (1). (CIS #42, MAC #42, LAU #29) The upper temporal fenestra is bounded by the postorbital, parietal and squamosal (Laurin & Reisz, 1995)

(65) Lower lateral temporal opening (fenestra or emargination): absent (0); present (1). (CIS #43, MAC #43, modified from LAU #30)

(66) Lower temporal fenestra or emargination shape: bounded ventrally, forming a fenestra (0); unbounded ventrally, forming an emargination (1). (CIS #44, MAC #44, modified from LAU #30)

(67) Lower temporal fenestra height: narrow dorsoventrally (0); tall dorsoventrally (1). (CIS #169)

(68) Postorbital contribution to lateral temporal opening (fenestra or emargination): present (0); absent (1). (CIS #45, MAC #45, LAU #20)

(69) Quadratojugal contribution to lateral temporal opening (fenestra or emargination): absent (0), present (1). (CIS #46, MAC #46, LAU #16)

(70) Ventral margin of skull region posterior to orbit: expanded below ventral extent of maxilla (0); rectilinear (1); emarginated (2). (CIS #47, MAC #47, LAU #32)

(71) Lateral surface of quadrate: covered by squamosal and quadratojugal (0); not covered (1). (CIS #48, MAC #48, LAU #34)

(72) Quadrate anterior process: extends anteriorly for at least 50% the length of the quadrate ramus (0); extends anteriorly for less than 50% the length of the quadrate ramus (1). (CIS #49, MAC #50, modified from LAU #49)

(73) Quadrate condyle articular surfaces: strongly convex, anteroposteriorly longer than they are wide (0); nearly flat, anteroposteriorly shorter than they are wide (1). (CIS #50, MAC #51, modified from LAU #50)

(74) Posterior margin of skull roof: roughly straight (0); with a single, median embayment (1); embayed bilaterally (2). (CIS #56, MAC #58, MOD99 #12)

(75) Temporal notch: present (0); absent (1). (CIS #57, MAC #59, MUL #134)

(76) Temporal depression associated with posterolateral excavation: restricted to the posterior half of the cheek (0); closely approaches the orbital margin (1). (CIS #58, MAC #60, MUL #135)

(77) Orbit exposure predominantly: laterodorsal (0); lateral (1); dorsal (2). (CIS #184)

(78) Posterolateral edge of skull roof: protruding, spine-like (0); does not protrude (1). (CIS #189)

(79) Occiput: low (0); high (1). (CIS #190)

(80) Alar flange of the vomer: absent (0); present (1). (CIS #62, MAC #64, TSU #50)

(81) Vomerine dentition: shagreen-like, multiple rows (0); concentrated along one or a double anteroposterior ridge (1). (CIS #185)

(82) Single large tooth on anteriormost end of vomer: absent (0); present (1). (CIS #167)

(83) Palatine dentition: shagreen-like (0); concentrated along one diagonal ridge (1). (CIS #186)

(84) Choana: parallel to maxilla; palatine forms its posterior edge only (0); curved posteromedially; palatine forms its posterior and part of its lateral edge (1). (CIS #61, MAC #63, LAU #40)

(85) Interpterygoid vacuity anterior extent: reaches beyond posterior border of palatine (0); reaches level of palatine or less (1). (CIS #59, MAC #61, modified from REI07 #127)

(86) Anterior shape of interpterygoid vacuity: acuminate (0); rounded (1). (CIS #60, MAC #62)

(87) Arcuate flange of pterygoid: present (0); absent (1). (CIS #63, MAC #65, LAU #42)

(88) Cranio-quadrate space: small, quadrate ramus of pterygoid and paraoccipital process of opisthotic converge posterolaterally (0); large, quadrate ramus of pterygoid and paraoccipital process of opisthotic are parallel to each other (1). (CIS #64, MAC #66, LAU #43)

(89) Pterygoid anterior extent: reaches level of posterior end of choana (0); posterior to choana (1). (CIS #65, MAC #67, LAU #44)

(90) Transverse flange of the pterygoid: large, approaches cheek, a noticeable lateral projection (0); small, does not approach cheek (1). (CIS #66, MAC #68, modified from LEE97 #19)

(91) Transverse flange of pterygoid orientation: directed posterolaterally or transversely (0); directed anterolaterally (1); directed anteriorly (2). (CIS #67, MAC #69, modified from LAU #45)

(92) Transverse flange of pterygoid dentition: present (0); absent (1). (CIS #68, MAC #70, modified from LAU #45)

(93) If dentition is present on the transverse flange of the pterygoid, it is a: shagreen of very small teeth, no ventral ridge (0); single row of large teeth, no ventral ridge (1). (Modified from CIS #69, MAC #71, modified from LAU #46)

(94) Quadrate flange of pterygoid dentition: absent (0); present (1). (CIS #70, MAC #72)

(95) Quadrate ramus of pterygoid: merges smoothly into transverse flange without distinctive excavation (0); deep excavation on posterolateral surface (1). (CIS #71, MAC #73)

(96) Quadrate ramus of pterygoid relation to trasverse flange: not continuous with transverse flange (0); continuous with transverse flange, forming a ridge (1). (CIS #72, MAC #74)

(97) Lateral pocket on the pterygoid found between quadrate ramus and transverse flange: absent (0); present (1). (CIS #73, MAC #75)

(98) Ectopterygoid: present (0); absent (1). (CIS #74, MAC #76)

(99) If ectopterygoid is present, ectopterygoid dentition is: present (0); absent (1). (CIS #75, MAC #77, modified from LAU #48)

(100) If ectopterygoid is present, its relationship to transverse flange: distal to transverse flange, does not contribute to lateral portion of flange (0); makes contact with lateral portion of transverse flange (1). (CIS #76, MAC #78, modified from DEB96 #33)

(101) Suborbital opening on the palate: absent (0); present (1). (CIS #77, MAC #79, modified from LAU #49)

(102) Basicranial articulation: kinetic/synovial (0); sutured and/or immobile (1). (CIS #78, MAC #80, LEE97 #2)

(103) Length of basicranial articulation: restricted to anterolateral margin of the parasphenoid (0); extends over much of length of main body of parasphenoid (1). (CIS #79, MAC #81, DEB96 #36)

(104) Parasphenoid pocket for cervical musculature (one or two): present (0); absent (1). (CIS #80, MAC #82, LAU #50)

(105) If a parasphenoid pocket for cervical musculature is present it is represented by: a single median pocket (0); two pockets (1). (CIS #81, MAC #83)

(106) Parasphenoid wings; present, parasphenoid broader posteriorly than long (0); absent, parasphenoid narrower posteriorly than long (1). (CIS #82, MAC #84, modified from LAU #51)

(107) Cultriform process: present (0); absent (1). (CIS #83, MAC #85, modified from LAU #52)

(108) If cultriform process is present, it is: longer than the body of the parasphenoid (0); shorter than the body of the parasphenoid (1). (CIS #84, MAC #86, modified from LAU #52)

(109) Parasphenoid teeth: absent (0); present (1). (CIS #85, MAC #87, modified from LAU #53)

(110) If parasphenoid teeth are present, they are found: in rows (0); as a shagreen (1). (CIS #86, MAC #88, modified from LAU #53)

(111) Supraoccipital: plate-like, with no sagittal crest (0); constricted at midline, forming sagittal crest (1); plate-like, with a saggital crest (2). (CIS #87, MAC #89, modified from DEB97 #56)

(112) Paroccipital process: vertically broad (0); anteroposteriorly expanded (1); narrow (2); tubular, composed of opisthotic (3). (CIS #88, MAC #90, LAU #56)

(113) Paroccipital process orientation: directed primarily laterally (0); oriented obliquely, at an angle of at least 45 degrees from the horizontal plane of the skull (1). (CIS #89, MAC #91, DEB96 #56)

(114) Sutural contact between paroccipital process and dermatocranium: absent (0); present (1). (CIS #90, MAC #92, modified from LAU #57)

(115) Otic trough in ventral flange of opisthotic: absent (0); present (1). (CIS #91, MAC #93, LAU #58)

(116) Medial wall of inner ear (made of prootic): unossified (0); ossified with acoustic nerve foramina (1). (CIS #92, MAC #94, LAU #59)

(117) Post-temporal fenestra: absent (0); present (1). (CIS #93, MAC #95, modified from DEB97 #59)

(118) If post-temporal fenestra is present, it is: small, diameter less than the diameter of foramen magnum (0); large, diameter at least equal to foramen magnum (1). (CIS #94, MAC #96, modified from DEB97 #59)

(119) Osseous contact between basioccipital and basisphenoid: present (0); absent (1). (CIS #95, MAC #97, LAU #61)

(120) Occipital condyle shape: transversely broad (0); reniform to circular (1). (CIS #96, MAC #98, LAU #62)

(121) Ventral exposure of basioccipital: contributes extensively to ventral surface of the braincase (0); restricted to condylar region (1). (CIS #97, MAC #99, DEB #37)

(122) Ventral braincase tubera: absent (0); present (1). (CIS #98, MAC #100, modified from DEB97 #65 & LAU #63)

(123) If ventral braincase tubera are present, they are: restricted to basioccipital (0); very large and restricted to basisphenoid (1). (CIS #99, MAC #101, modified from DEB97 #65 & LAU #63)

(124) Lateral flange of exoccipital: absent (0); present (1). (CIS #100, MAC #102, LAU #64)

(125) Stapes: robust, greatest depth exceeding one-third of total length (0); slender, length at least four times depth (1). (CIS #101, MAC #103, modified from DEB97 #45)

(126) Stapedial dorsal process: ossified (0); unossified (1). (CIS #102, MAC #104, LAU #69)

(127) Stapedial shaft morphology: elongated and narrow, tapers distally (0); short and robust, expands distally (1). [New character]

(128) Stapedial shaft-dorsal process contact: absent (0); present (1). Refers to a continuous ridge extending between the anterior stapedial shaft and the dorsal process. [New character]

(129) Presence of size related heterodonty on the anterior 2/3 of the dentary: present (0); absent (1). (CIS #36, MAC #36)

(130) Bulbous marginal teeth: absent (0); present (1). (CIS #168) Teeth are considered to be bulbous when the largest teeth have maximum widths that are equal to or more than their maximum height (MacDougall et al., 2017)

(131) Coronoid number: two or three (0); one (1). (CIS #111, MAC #113, LAU #74)

(132) Coronoid process: low (0), high (1). (CIS #117, MAC #119, modified from LAU #79)

(133) If coronoid process is high, it is: composed of coronoid only (0); composed of dentary and coronoid (1). (CIS #118, MAC #120, modified from LAU #79)

(134) Splenial: contributes to mandibular symphysis (0); excluded from mandibular symphysis (1). (CIS #119, MAC #121, LAU #80)

(135) Foramen intermandibularis: an anterior symphysial foramen (0); an anterior symphysial foramen and a posterior foramen (1). (CIS #105, MAC #106, modified from LAU #69)

(136) If there are two intermandibular foramina the posterior foramen is located: anterior to coronoid process (0); posterior to or at level of coronoid process (1). (CIS #106, MAC #107, modified from LAU #69)

(137) Meckelian fossa anteroposterior length: long, occupies at least 25% of lower jaw length (0); short, occupies less than 25% of lower jaw length (1). (CIS #108, MAC #109, modified from LAU #71)

(138) Meckelian fossa orientation: faces mediodorsally (0); faces dorsally (1). (CIS #107, MAC #108, modified from LAU #70)

(139) Surangular length: extends beyond coronoid eminence (0); does not extend beyond coronoid eminence (1). (CIS #109, MAC #110, LAU #72)

(140) Accessory lateral shelf on surangular anterior to articular region: absent (0); present (1). (CIS #110, MAC #111, LAU #73)

(141) Paired foramina on the anteroposterior midline of the surangular: absent (0); present (1). (CIS #104, MAC #112)

(142) Prearticular extends: beyond the coronoid eminence (0); does not extend beyond coronoid eminence (1). (CIS #112, MAC #114, modified from LAU #75)

(143) Retroarticular process: present (0); absent (1). (CIS #113, MAC #115, modified from Laurin & Reisz, 1995 #76)

(144) If present, the retroarticular process is: small and narrow (0); transversely broad, dorsally concave (1). (CIS #114, MAC #116, modified from LAU #76)

(145) If present, the retroarticular process is composed of: articular body (0); three or more elements (articular, prearticular, angular and surangular) (1). (CIS #115, MAC #116, modified from LAU #76)

(146) Lateral shelf on articular region: absent (0); present (1). (CIS #116, MAC #118, modified from LAU #78)

(147) Jaw articulation position: posterior to occiput (0); even with occiput (1); anterior to occiput (2). (CIS #51, MAC #52, LAU #36)

(148) Presacral vertebral count: more than twenty (0); twenty or less (1). (CIS #120, MAC #122, LAU #81)

(149) Axial centrum orientation: in plane of axial skeleton (0); sloping anterodorsally (1). (CIS #121, MAC #123, LAU #121)

(150) Atlantal epipophysis: possesses epipophysis (0); lacks epipophysis (1). (CIS #122, MAC #124, modified from MOD99 #126)

(151) Axial intercentrum: with rounded anteroventral edge (0); with strong anterior process (1). (CIS #123, MAC #125, LAU #84)

(152) Atlantal pleurocentrum and axial intercentrum: separate elements (0); attached or fused (1). (CIS #124, MAC #126, LAU #85)

(153) Trunk neural arches: swollen (0); narrow (1). (CIS #125, MAC #127, modified from LAU #86)

(154) Ventral surface of anterior pleurocentra: ventral surface of vertebral centra uniform (0); ventral surface of vertebral centra bearing an excavation on either side of the midline, coupled with a flattened median crest between them (1). (CIS #126, MAC #128, modified from LAU #87)

(155) Posterior dorsal neural spine orientation: approximately vertical (0); posteriormost one or two dorsal neural spines anterodorsally inclined (1); several posterior neural spines anterodorsally inclined (2). (CIS #176)

(156) Number of sacral vertebrae: one (0); two (1); three or more (2). (CIS #127, MAC #129, LAU #88)

(157) Sacral rib distal overlap: broad with narrow gap between ribs (0); small or absent with wide gap between ribs (1). (CIS #128, MAC #130, LAU #89)

(158) Transverse process or ribs: present only on a few anterior caudals (0); present on at least thirteen caudals (1). (CIS #129, MAC #131, LAU #90)

(159) Anterior caudal rib size: elongate and extend posteriorly to the end of the next vertebra (0); curve posteriorly but do not extend to the end of the next vertebrae (1); straight, with no posterior curvature (2). (CIS #130, MAC #132)

(160) Caudal hemal arches: wedged between centra (0); attached to anterior centrum (1). (CIS #131, MAC #133, LAU #91)

(161) Interclavicle: diamond-shaped (0); T-shaped, with long, slender lateral processes (1). (CIS #132, MAC #134, LAU #92)

(162) Interclavicle attachment for clavicle: ventral sutural area (0); anteriorly directed groove (1); tightly sutured into plastron (2). (CIS #133, MAC #135, LAU #93)

(163) Cleithrum: present (0); absent (1). (CIS #134, MAC #136, modified from LAU #94)

(164) If cleithrum is present, it: caps scapula anterodorsally (0); does not cap scapula at all (1). (CIS #135, MAC #137, modified from LAU #94)

(165) Scapula: broad and low (0); narrow and high (1). (CIS #136, MAC #138, modified from LAU #96) A low scapula has a height equal to or less than its anteroposterior length (MacDougall et al., 2017)

(166) Supraglenoid foramen: present (0); absent (1). (CIS #137, MAC #139, LAU #97)

(167) Glenoid: helical, composed of a single facet (0); bipartite, composed of two facets (1). (CIS #138, MAC #140, modified from LAU #98)

(168) Acromion: absent (0); present (1). (CIS #139, MAC #141, LAU #99)

(169) Sternum mineralization: absent (0); present (1). (CIS #140, MAC #142, LAU #100)

(170) Supinator process orientation: strongly angled relative to shaft (0); parallel to shaft (1). (CIS #141, MAC #143, modified from LAU #101)

(171) If supinator process is parallel to shaft it is: separated from it by a groove (0); not separated from shaft (1). (CIS #142, MAC #144, modified from LAU #101)

(172) Ectepicondylar foramen: only groove present (0); groove and foramen present (1); only foramen present (2); both absent (3). (CIS #143, MAC #145, LAU #102)

(173) Entepicondylar foramen: present (0); absent or not fully enclosed (1). (CIS #144, MAC #146, LAU #103)

(174) Humerus: with robust heads and a short shaft (0); short and robust, without a distinct shaft (1); slender with long shaft (2). (CIS #145, MAC #147, modified from LAU #104) A short shaft has a proximodistal length equal to or less than the mediolateral width of the heads (MacDougall et al., 2017)

(175) Olecranon process: present (0); absent (1). (CIS #146, MAC #148, modified from LAU #105)

(176) If present, the olecranon process is: large, with articular facet of ulna facing medially (0); small, with articular facet of ulna facing proximally (1). (CIS #147, MAC #149, modified from LAU #105)

(177) Manual phalangeal formula: 2 3 4 5 3 (0); 2 3 4 4 3 (1); 2 3 3 3 3 or less (2). (CIS #148, MAC #150, LAU #106)

(178) Dorsolateral shelf on iliac blade: absent (0); present (1). (CIS #149, MAC #151, LAU #107)

(179) Iliac blade: low, with long posterodorsal process that extends beyond the posterior edge of the iliac body (0); dorsally expanded, distally flaring, the posterodorsal process does not extended beyond the posterior edge of the iliac body (1). (CIS #150, MAC #152, LAU #108)

(180) Acetabular buttress: small, overhangs acetabulum only moderately (0); large, overhangs acetabulum strongly (1). (CIS #151, MAU #153, LAU #109)

(181) Oblique ventral ridge of femur (adductor crest): present (0); absent (1). (CIS #152, MAU #154, LAU #110)

(182) Femoral proximal articulation: anteroposteriorly long (0); round (1). (CIS #153, MAU #155, LAU #111)

(183) Greater trochanter of femur: absent (0); present on posterior edge of femur (1). (CIS #154, MAC #156, LAU #112)

(184) Femoral shaft: short and broad (0); long and slender (1). (CIS #155, MAC #157, LAU #113) A short and broad femoral shaft has a proximodistal length equal to or less than the mediolateral width of the distal head in ventral view (Laurin & Reisz, 1995).

(185) Astragalus: absent (0); present (1). (CIS #156, MAC #158, modified from LAU #115)

(186) If astragalus is present, it: incorporates incompletely fused tibiale, intermedium, and perhaps centrale 4 (0); is without traces of compound origin (1). (CIS #157, MAC #159, modified from LAU #115)

(187) Tibio-astragalar joint: flat (0); tibial ridge fits into astragalar groove (1). (CIS #158, MAC #160, LAU #116)

(188) Astragalus and calcaneum: separate (0); sutured or fused (1). (CIS #159, MAC #161, LAU #117)

(189) Medial pedal centrale: present (0); absent (1). (CIS #160, MAC #162, LAU #118)

(190) Number of distal tarsals: five (0); four or less (1). (CIS #161, MAC #163, LAU #119)

(191) Metapodials: not overlapping (0); overlapping (1). (CIS #162, MAC #164, LAU #121)

(192) Pedal phalangeal formula: 2 3 4 5 4 (0); 2 3 4 4 3 (1); 2 3 3 4 3 or less (2). (CIS #163, MAC #165, LAU #122)

(193) Ratio between length of metatarsal I to length of metatarsal IV: at least 0.5 (0); less than 0.5 (1). (CIS #164, MAC #166, LAU #123)

Deleted characters:

- CIS #55: Dorsal dermal ossifications: absent (0); present (1). (CIS #55, MAC #57, LAU #124)
- CIS #103: Marginal dentition: single cusp (0); two to seven cusps (1); more than seven cusps (2).
- CIS #172: Squamosal contribution to lower temporal bar: absent (0); present (1).
